# Transposed Maxillary Canines: Narrative Review with Clinical Case Report

**DOI:** 10.3390/dj13060251

**Published:** 2025-06-05

**Authors:** Teresa Pinho, Rui Amaral

**Affiliations:** 1 UNIPRO—Oral Pathology and Rehabilitation Research Unit, University Institute of Health Science (IUCS), 4585-116 Gandra, Portugal; a29519@alunos.cespu.pt; 2 UMIB—Multidisciplinary Biomedical Research Unit, Abel Salazar Institute of Biomedical Sciences (ICBAS), University of Porto, 4050-313 Porto, Portugal

**Keywords:** cuspid, tooth eruption, ectopic, maxilla, orthodontics

## Abstract

Permanent maxillary canines are key teeth from both functional and aesthetic perspectives. Tooth transposition, a rare anomaly where two permanent teeth exchange positions within the same quadrant, poses significant diagnostic and therapeutic challenges. This study aimed to identify the clinical conditions guiding the decision to correct or maintain maxillary canine transposition through a narrative review of the literature, complemented by a detailed clinical case. Following PRISMA guidelines, a search of PubMed, ScienceDirect, Cochrane Library, EBSCO, and Scopus databases (2014–May 2024) yielded 19 relevant studies. This review found no consensus on treatment protocols, reinforcing the need for individualized decision-making based on patient characteristics, anatomical constraints, and clinician expertise. While orthodontic correction can yield favorable aesthetic outcomes, it requires meticulous planning and biomechanical control. Conservative approaches are often indicated in early-diagnosed or anatomically complex cases. The clinical case illustrates the complexity of managing the transposition, specifically of the maxillary canine into the central incisor position, emphasizing the importance of early diagnosis, digital planning, and a multidisciplinary approach for functional and aesthetic success.

## 1. Introduction

Permanent maxillary canines are vital due to their distinct anatomy, position in the arch, and roles in occlusion, guidance, and aesthetics. They provide lateral guidance, support facial structure, and contribute to arch stability, making them key functional and aesthetic teeth [1]. However, their indispensability is debated, as alternative occlusal schemes like group function can sometimes compensate for their absence, particularly in restorative or post-orthodontic contexts [2]. However, their long eruption pathway makes them prone to ectopic eruption or impaction, with a prevalence of 1–3% [3].

A rarer anomaly is tooth transposition, where two permanent teeth exchange positions within the same quadrant, defined as maxillary canine transposition (MCT). This condition often coexists with other anomalies, such as agenesis, conical shapes, inclusion, or malformations of adjacent teeth [4]. Transposition predominantly occurs unilaterally, is more common in females, and affects the upper arch more frequently than the lower. It is classified as either complete (both crown and root transposed) or incomplete (only the crown transposed while the root apex remains in position) [4].

The most frequent transpositions involve maxillary canines and adjacent teeth, including the first premolar or lateral incisor. Five common maxillary transpositions are described as follows: between the canine and first premolar (Mx.C.P1), the canine and lateral incisor (Mx.C.I2), the canine and first molar (Mx.C.M1), central and lateral incisors (Mx.I2.I1), and the canine and central incisor (Mx.C.I1) [4].

The etiology of MCT and impacted maxillary canines (IMC) remains uncertain, with theories suggesting factors such as the prolonged retention of deciduous teeth, obstacles in the eruptive path (e.g., supernumerary teeth or odontomas), trauma, genetic predisposition, and arch crowding [4,5]. Cone beam computed tomography (CBCT) is currently the most effective tool for diagnosing and planning treatment for these cases [6].

A multidisciplinary approach is often required in maxillary canine transposition cases. Depending on the anatomical and clinical conditions, the goal may be either to correct the transposition or to maintain it. Each option may involve different procedures, such as orthodontic traction, the extraction of structurally compromised teeth, implant placement, prosthetic reshaping, or orthodontic compromises, like canine substitution. Accurate diagnosis and individualized treatment planning are essential for successful outcomes [4,7].

This study aims to deepen the understanding of the variables that influence the selection of appropriate treatment plans for transposed maxillary canines. Specifically, it seeks to identify the clinical conditions under which correcting or maintaining maxillary canine transposition (MCT) is the most suitable approach. The inclusion of a highly complex clinical case featuring the transposition of the canine into the central incisor position adds valuable clinical insight and reinforces the importance of integrating real-world evidence into reviews.

## 2. Data Description

### 2.1. Study Design

This study integrates a narrative review, following established clinical guidelines, along with the detailed description of a clinical case report to illustrate clinical decision-making in maxillary canine transposition (MCT).

### 2.2. Review Guidelines

This narrative review was conducted following the Preferred Reporting Items for Narrative Reviews and Meta-analysis (PRISMA) 2020 guidelines. The study protocol was registered in the PROSPERO database (CRD42024612605).

### 2.3. Selection Criteria

To be included in this study, articles had to meet the following conditions:

Inclusion criteria:

- Articles addressing MCT;
- Articles published in English between 2014 and 2024;
- Articles involving human subjects;
- Articles classified as retrospective, case-control, cross-sectional studies, or case reports.

Exclusion criteria:

- Encompassed articles involving participants without transposed maxillary canines;
- Articles lacking relevant details on MCT.

### 2.4. Eligibility Criteria

The PICOS (Population, Intervention, Comparison, Outcomes, and Study design) strategy was employed to formulate the research question: “Under which conditions should maxillary canine transposition be corrected or maintained?” (Table 1).

### 2.5. Search Strategy

A comprehensive bibliographic search was conducted across PubMed, ScienceDirect, Cochrane Library, EBSCO, and Scopus databases, covering publications from 2014 to May 2024. Specific keywords and MeSH terms utilized in this search are summarized in Table 2.

### 2.6. Selection of Articles and Data Collection

**Figure 1:**
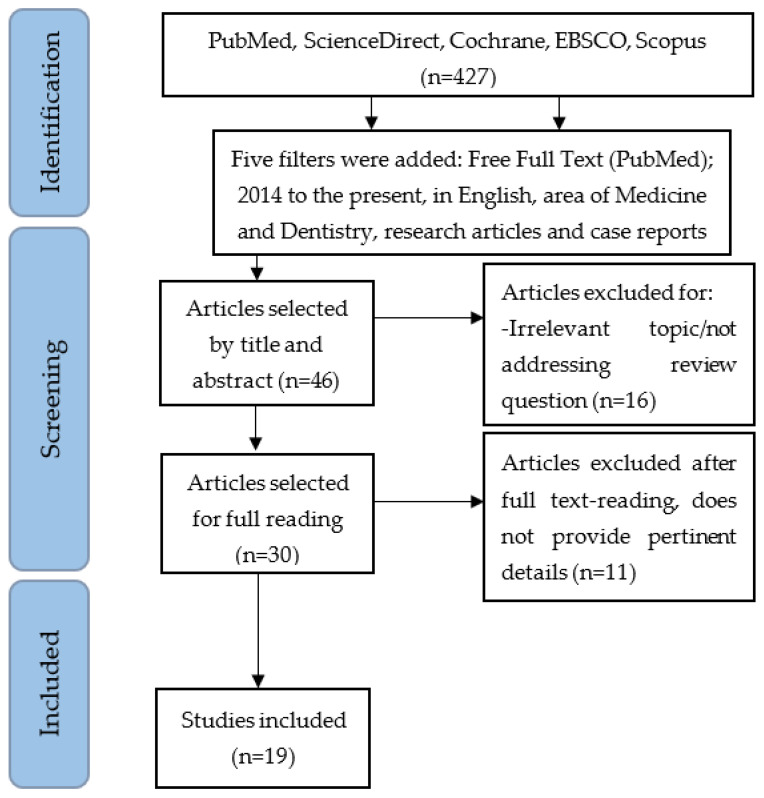
Flowchart of the research strategy used in this study (PRISMA).

Initially, titles and abstracts from potentially relevant articles were screened. Eligible articles underwent full-text analysis for inclusion, with relevant data extracted and systematically summarized in Table 3 and Table 4. The selection process is detailed through a *PRISMA* flowchart (Figure 1).

### 2.7. Clinical Case

A clinical case with maxillary canine–central incisor transposition (Mx.C.I1) was selected to exemplify the treatment decisions informed by the narrative review. It included a detailed clinical assessment, panoramic radiography, cone beam computed tomography (CBCT), and specific linear and angular measurements. Clinical documentations, including pre- and post-treatment intraoral photos and imaging data, are illustrated in Figures 7–25.

## 3. Results

### 3.1. Selection of Articles

The bibliographic search identified a total of 427 articles. After analyzing titles and abstracts, 30 articles were selected for full-text evaluation. Following detailed analysis, 11 articles were excluded due to insufficient relevant information. Ultimately, 19 articles were included in the review, as summarized in Figure 1 (PRISMA flowchart) [6,7,8,9,10,11,12,13,14,15,16,17,18,19,20,21,22,23,24]. Data extracted from the included case reports and retrospective studies are presented in Table 3 and Table 4, respectively. The quality assessment of the included studies was conducted by utilizing the ROBINS-I tool for observational studies and the Joanna Briggs Institute critical appraisal checklist for case reports.

#### 3.1.1. Quality Assessment

Table 3 and Table 4 summarize the quality assessment of the included studies.

**Table 3:** Risk of bias in non-randomized studies of interventions (ROBINS-I) [25,26].

Risk of Bias Domains							
Author and Year of Publication	Bias Due to Confounding	Bias in Selection of Participants into the Study	Bias in Classification of Interventions	Bias due to Deviations from Intended Interventions	Bias due to Missing Data	Bias in Measurement of Outcomes	Bias in Selection of the Reported Result
Maspero C. et al. (2016) [8]	Low	Low	Low	Low	Moderate	Low	Low
Finkelstein T. et al. (2020) [9]	Low	Low	Low	Low	Moderate	Low	Low

**Table 4:** Risk of bias of case reports by JBI critical appraisal checklist [25,26].

Checklist	Gebert TJ. et al. (2014) [11]	Di Palma E. et al. (2015) [12]	Teresa DM. et al. (2015) [13]	Hsu YL. et al. (2016) [14]	Lorente T. et al. (2016) [15]	Potrubacz MI. et al. (2016) [8]	Nabbout F. et al. (2017) [16]	Lara MS. et al. (2018) [17]	Matsumoto MAN. et al. (2018) [18]
1. Were patient’s demographic characteristics clearly described?	Yes	Yes	Yes	Yes	Yes	Yes	Yes	Yes	Yes
2. Was the patient’s history clearly described and presented as a timeline?	Yes	Yes	Yes	Yes	Yes	Yes	Yes	Yes	Yes
3. Was the current clinical condition of the patient on presentation clearly described?	Yes	Yes	Yes	Yes	Yes	Yes	Yes	Yes	Yes
4. Were diagnostic tests or assessment methods and the results clearly described?	Yes	Yes	Yes	Yes	Yes	Yes	Yes	Yes	Yes
5. Was the intervention(s) or treatment procedure(s) clearly described?	Yes	Yes	Yes	Yes	Yes	Yes	Yes	Yes	Yes
6. Was the post-intervention clinical condition clearly described?	Yes	Yes	Yes	Yes	Yes	Yes	Yes	Yes	Yes
7. Were adverse events (harms) or unanticipated events identified and described?	No	No	Yes	No	Yes	No	No	Yes	No
8. Does the case report provide takeaway lessons?	Yes	Yes	Yes	Yes	Yes	Yes	Yes	Yes	Yes
Overall appraisal:	Include	Include	Include	Include	Include	Include	Include	Include	Include
Checklist	de Souza RM. et al. (2020) [7]	Pedalino A. et al. (2020) [19]	Lorente C. et al. (2020) [6]	Mereani S. et al. (2022) [20]	Paixão MPM. et al. (2023) [21]	QasemAl-Gazzawi AM. et al. (2023) [22]	Pithon MM. et al. (2023) [23]		Andrade EC. et al. (2023) [24]
1. Were patient’s demographic characteristics clearly described?	Yes	Yes	Yes	Yes	Yes	Yes	Yes		Yes
2. Was the patient’s history clearly described and presented as a timeline?	Yes	Yes	Yes	Yes	Yes	Yes	Yes		Yes
3. Was the current clinical condition of the patient on presentation clearly described?	Yes	Yes	Yes	Yes	Yes	Yes	Yes		Yes
4. Were diagnostic tests or assessment methods and the results clearly described?	Yes	Yes	Yes	Yes	Yes	Yes	Yes		Yes
5. Was the intervention(s) or treatment procedure(s) clearly described?	Yes	Yes	Yes	Yes	Yes	Yes	Yes		Yes
6. Was the post-intervention clinical condition clearly described?	Yes	Yes	Yes	Yes	Yes	Yes	Yes		Yes
7. Were adverse events (harms) or unanticipated events identified and described?	Yes	No	Yes	Yes	No	No	No		Yes
8. Does the case report provide takeaway lessons?	Yes	Yes	Yes	Yes	Yes	Yes	Yes		Yes
Overall appraisal:	Include	Include	Include	Include	Include	Include	Include		Include

The methodological quality of included retrospective studies was assessed using the ROBINS-I tool, while case reports were evaluated using the JBI critical appraisal checklist. Two reviewers independently appraised quality across the following seven bias domains: confounding, participant selection, intervention classification, deviations from intended interventions, missing data, outcome measurement, and selection of reported results. Maspero C. et al. (2016) [9] and Finkelstein T. et al. (2020) [10] were identified as having a moderate risk of bias. All included case reports demonstrated minimal risk of bias. Table 5 and Table 6 summarize the data and outcomes of the included case reports and retrospective studies, respectively.

**Table 5:** Data and outcomes from case reports.

Authors and Year of Publication	Population	Transposition	Complete or Incomplete	Right, Left, or Bilateral	Vestibular or Palatal	Sagittal	Treatment	Side Effects
						*Relation*		
						(*Before*)		
Gebert TJ. et al. 2014 [11]	♀ 12 YO	Mx.C.I2	I	L	V (erupted)	R: Cl I L: Cl II	Corrected	-
Di Palma E. et al. 2015 [12]	♀ 7 YO	Mx.C.P1	C	B	V (erupted)	Cl II div 2 Sk I	Maintained	-
Teresa DM. et al. 2015 [13]	♂ 12 YO	Mx.C.P1	C	R	V (impacted)	Cl I	Corrected	-Tooth #13 showed slight gingival recession
Hsu YL. et al. 2016 [14]	♀ 12 YO	Mx.C.I2	C	L	V (erupted)	Cl II div 1 Sk II	Corrected	-
Lorente T. et al. 2016 [15]	1: ♂ 12 YO 2: ♀ 15 YO	1:Mx.C.I2 2:Mx.C.I2	1: I 2: C	1: B 2: L	1:V (impacted) 2:V (impacted)	1: Cl I 2: Cl I	1: Corrected 2: Corrected	-Teeth #12 and #22 showed root resorption before and after treatment
Potrubacz MI. et al. 2016 [8]	♀ 7 YO	Mx.C.P1	C	B	V (erupted)	Cl III Sk III	Maintained	-
Nabbout F. et al. 2017 [16]	1: ♀ 13 YO 2: ♀ 11 YO	1:Mx.C.P1 2:Mx.C.P1	1: C 2: C	1: L 2: R	1:V (erupted) 2:V (erupted)	1: Cl II Sk I 2: MSTP Sk I	1: Corrected 2: Corrected	-
Lara MS. et al. 2018 [17]	♀ 9 YO	Mx.C.I1	C	L	P (impacted)	Cl I Sk II	Maintained	-Slight root angulation of tooth #13, overall good root parallelism -Diminished root length due to orthodontic treatment before apex closure
Matsumoto MAN. et al. 2018 [18]	♀ 17 YO	Mx.C.I2	C	R	V (erupted)	Cl Ind Sk II	Corrected	-
de Souza RM. et al. 2020 [7]	♂ 9 YO	Mx.C.P1	C	R	P (impacted)	Cl II Sk II	Maintained	-Good root parallelism overall, except tooth #14 -Slight apical root resorption observed in maxillary anterior teeth
Pedalino A. et al. 2020 [19]	♀ 12 YO	Mx.C.I2	C	B	V (impacted)	Cl I Sk I	Corrected	-
Lorente C. et al. 2020 [6]	♂ 12 YO	Mx.C.P1	R: C L: I	B	V (erupted)	R: Cl II L: Cl I Sk II	Corrected	-Slight gingival recession on tooth #13
Mereani S. et al. 2022 [20]	♀ 14 YO	Mx.C.P1	C	B	V (erupted)	Cl I Sk I	Maintained	-Mucogingival issues on teeth #13 and #23
Paixão MPM. et al. 2023 [21]	♀ 11 YO	Mx.C.I1	C	R	P (impacted)	Cl I Sk I	Maintained	-
QasemAl-Gazzawi AM. et al. 2023 [22]	♀ 13 YO	Mx.C.I2	C	B	V (impacted)	Cl II Sk I	Corrected	-
Pithon MM. et al. 2023 [23]	♀ 15 YO	Mx.C.P1	C	B	V (erupted)	Cl I Sk I	Corrected	-
Andrade EC. et al. 2023 [24]	♂ 9 YO	Mx.C.I2	C	R	V (impacted)	Cl Ind	Corrected	-Decreased buccal cortical bone on teeth #11, #12, and #13 -Slight external root resorption on tooth #12 -Pulp necrosis, likely due to root movement on tooth #11
Pinho T., Amaral R. 2025	♀ 13 YO	Mx.C.I1	C	R	V (impacted)	Cl I	Maintained	-Total root resorption of tooth #11 due to the position of the ectopic canine

Abbreviations: Mx.C.I2: maxillary canine-lateral incisor transposition, Mx.C.P1: maxillary canine-first premolar transposition, Mx.C.I1: maxillary canine-central incisor transposition, C: complete, I: incomplete, L: left, B: bilateral, R: right, V: vestibular, P: palatal, Cl: angle class, Sk: skeletal class, Ind: indetermined, MSTP: mesial step terminal plane, #: tooth number.

**Table 6:** Data and outcomes from retrospective studies.

Authors and Year of Publication	Population	Transposition	Complete or Incomplete	Sagittal Relation (Before)	Treatment
Maspero C. et al. (2016) [9]	N = 20 between 8 and 12 YO (mean age 10 ± 0.3 YO);	15 Mx.C.P1	I	-	13 were Corrected 2 were Maintained
Finkelstein T. et al. (2020) [10]	N = 3.000:1.780 ♀ (59%) and 1.220 ♂ (41%) Ages: between 10 and 40 YO (mean age of 17.3 ± 9.3 YO)	6 Mx.C.P1 7 Mx.C.I2	-	All had Cl I	6 were Maintained 7 were Corrected

Abbreviations: Mx.C.P1: maxillary canine-first premolar transposition, Mx.C.I2: maxillary canine-lateral incisor transposition, I: incomplete, Cl: angle class.

#### 3.1.2. Data Analysis

**Figure 2:**
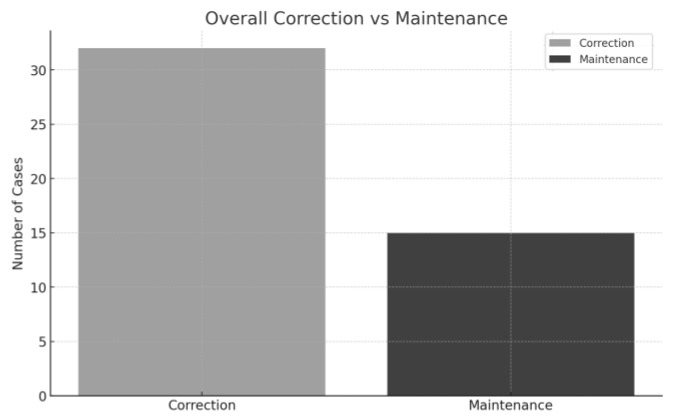
Overall correction and maintenance of MCT on the cases analyzed.

The analysis included a total of 48 cases of maxillary canine transposition (MCT), comprising 20 case reports and 28 cases from retrospective studies. Among the case reports, 13 patients (65%) underwent orthodontic correction of the transposition [6,11,13,14,15,16,18,19,22,23,24], while seven (35%) had the transposition maintained [7,8,12,17,20,21]. Of these, eight cases reported treatment-related side effects [6,7,13,15,17,20,21,24]. In the retrospective studies, 20 of the 28 cases (71.4%) were corrected [9,10], and 8 (28.6%) were maintained [9,10]. Overall, 33 cases (68.7%) underwent orthodontic correction, while 15 cases (31.3%) were maintained, indicating a general trend favoring correction (Figure 2).

**Figure 3:**
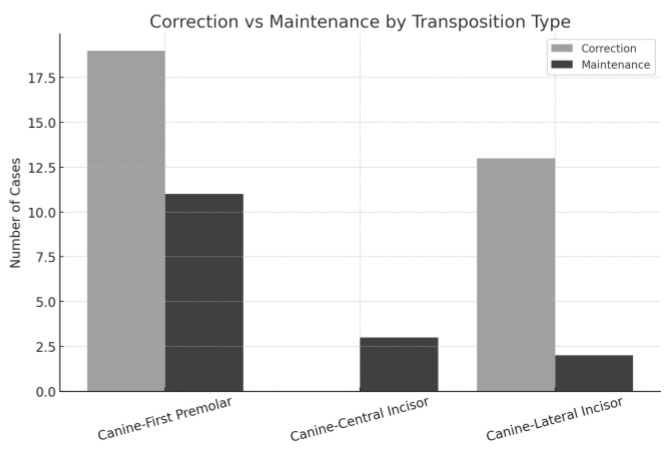
Correction and maintenance of MCT comparing the teeth affected on the cases analyzed.

Regarding the type of transposition, the most frequent was between the canine and first premolar (Mx.C.P1), with a correction rate of approximately 66.7% [6,9,10,13,16,23] and maintenance in 33.3% of cases [7,8,12,20]. Canine–lateral incisor transpositions (Mx.C.I2) showed the highest correction rate, with 86.7% corrected [10,11,14,15,18,19,22,24] and only 13.3% maintained [15]. In contrast, all cases of canine–central incisor transposition (Mx.C.I1) were maintained [17,21], reflecting the increased anatomical complexity and reduced feasibility of correction in these situations (Figure 3).

**Figure 4:**
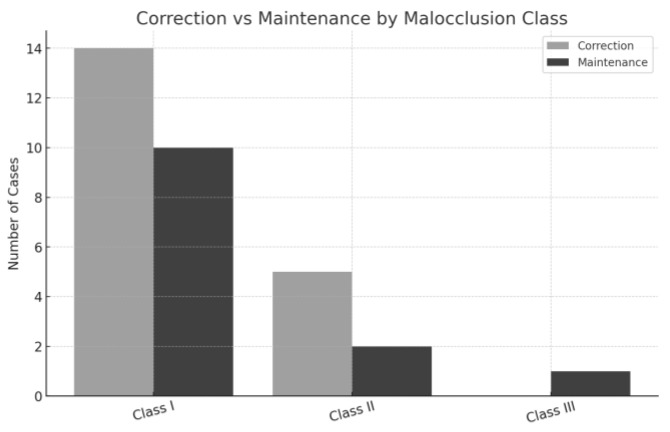
Correction and maintenance of MCT comparing malocclusions on the cases analyzed.

In terms of Angle’s malocclusion classification, Class II cases exhibited the highest correction rate (71.4%) [6,14,16,22], with 5 out of 7 cases corrected. Class I was the most prevalent, accounting for 24 cases (22 bilateral and 2 unilateral), with 58.3% corrected [10,11,13,15,18,19,22,23] and 41.7% maintained [7,10,12,17,20,21]. The single Class III case was maintained [8], and both cases with indeterminate or mesial step occlusion were corrected [16,18] (Figure 4).

**Figure 5:**
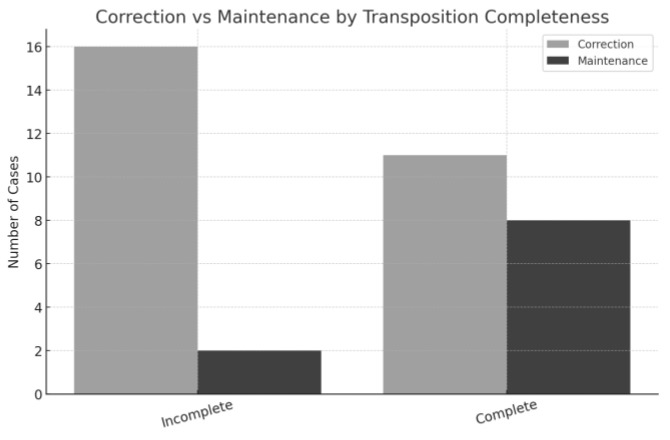
Correction and maintenance of MCT comparing complete and incomplete transpositions on the cases analyzed.

With respect to transposition completeness, incomplete transpositions showed a strong preference for correction, with 16 of 18 cases (89%) corrected [6,9,11,15] and only 2 maintained [9]. Complete transpositions presented a higher rate of maintenance, with 7 of 18 cases (38.9%) maintained [7,8,12,17,20,21] and 11 (61.1%) corrected [6,13,14,15,16,18,19,22,23,24] (Figure 5).

**Figure 6:**
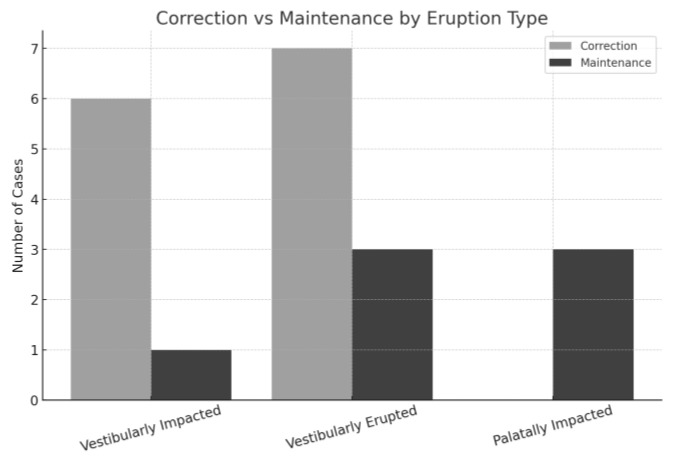
Correction and maintenance of MCT comparing eruption types on the cases analyzed.

Finally, regarding the eruption pattern of transposed canines, among the 20 case reports analyzed, 10 cases (50%) were classified as vestibularly erupted, seven (35%) as vestibularly impacted, and three (15%) as palatally impacted. Of the vestibularly impacted cases, six (85.7%) were corrected [13,14,15,16,19,22,24] and one (14.3%) maintained. In the vestibularly erupted group, seven cases (70%) were corrected [6,11,13,14,16,18,22,23] and three (30%) maintained [8,20]. Notably, all three palatally impacted cases (100%) were maintained [7,8,20], highlighting the complexity and surgical difficulty involved in such scenarios (Figure 6).

### 3.2. Clinical Case

**Figure 7:**
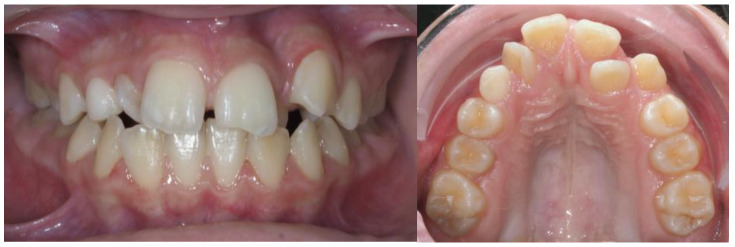
Initial intraoral frontal and occlusal photos.

**Figure 8:**
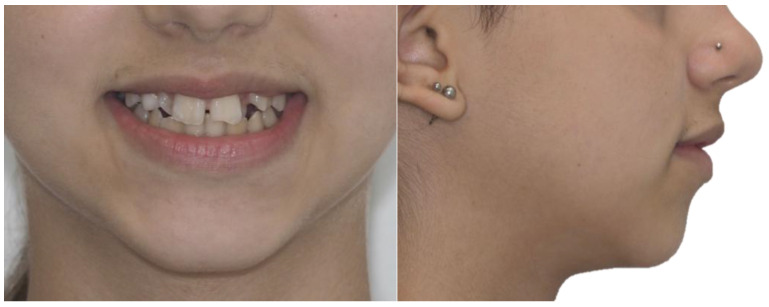
Initial smile and profile photos.

A 13-year-old Caucasian female presented with increased mobility of tooth #11. Clinical examination revealed full permanent dentition with a retained deciduous canine (#53), which was stable and showed no signs of mobility. The patient had bilateral Angle Class I molar relationships, moderate crowding, and an anterior diastema. On the contralateral side, ectopic eruption of tooth #22 was observed with palatal displacement, and tooth #23 appeared slightly buccally displaced. The persistence of tooth #53, despite the eruption of tooth #23, raised clinical suspicion of an impaction of tooth #13, especially given the absence of a palpable canine bulge in the buccal sulcus (Figure 7 and Figure 8).

**Figure 9:**
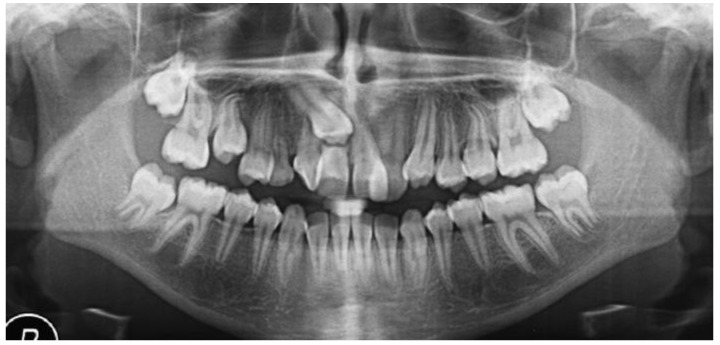
Panoramic radiograph showing transposition of tooth #13 over teeth #11 and #12.

**Figure 10:**
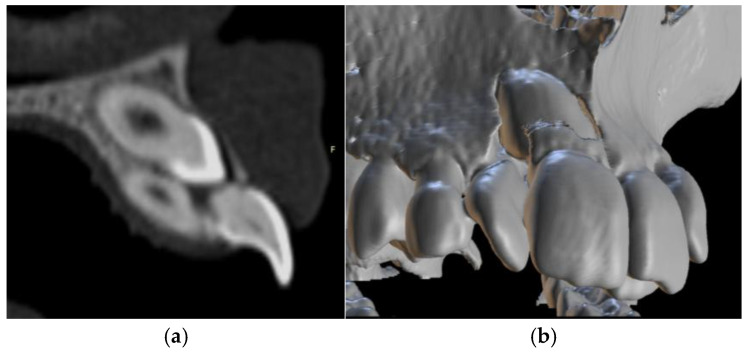
(**a**). CBCT image showing root resorption of tooth #11 and distal displacement of tooth #12 due to transposition of tooth #13; (**b**) transposed canine inside the root of tooth #11.

Radiographic assessment using panoramic imaging and CBCT confirmed a complex transposition involving tooth #13. The canine was ectopically positioned in the location of the maxillary central incisor (#11), overlapping both #11 and #12. The crown of tooth #13 was located near the cervical third of tooth #11, which exhibited complete root resorption. Additionally, tooth #12 showed severe displacement, with its root nearly horizontally oriented in a bucco-palatal direction, located behind the crown of the transposed canine (Figure 9 and Figure 10a,b).

**Figure 11:**
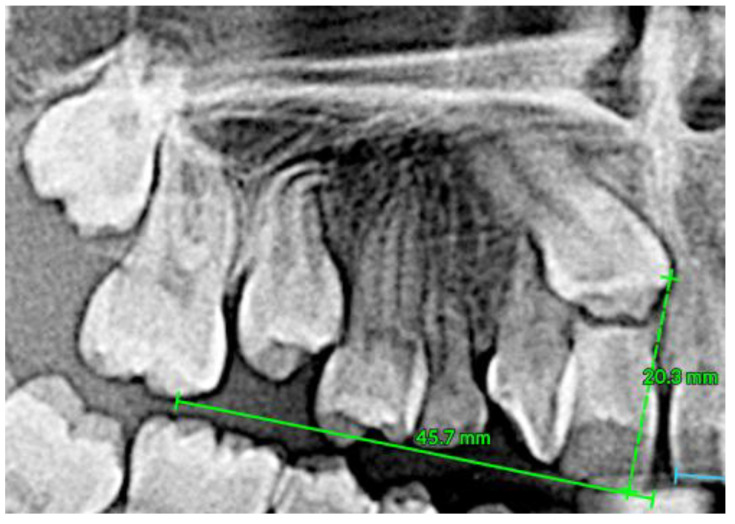
Distance d1 (distance from the maxillary canine cusp to the occlusal plane).

**Figure 12:**
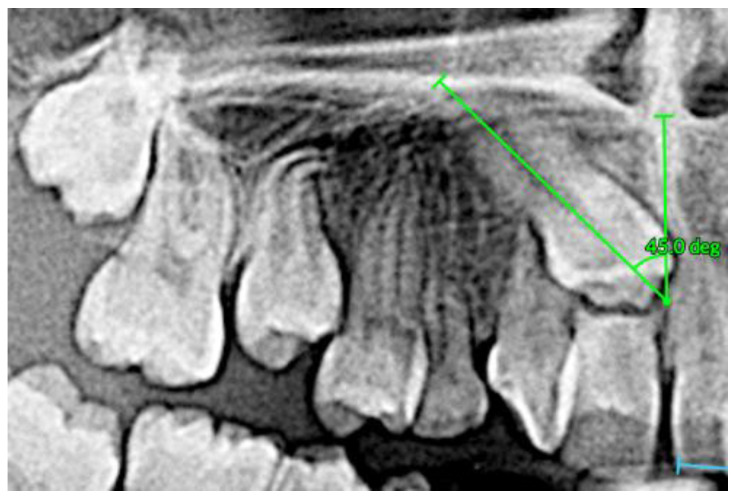
α Angulation (angle formed by the MC long axis and the interincisal line).

**Figure 13:**
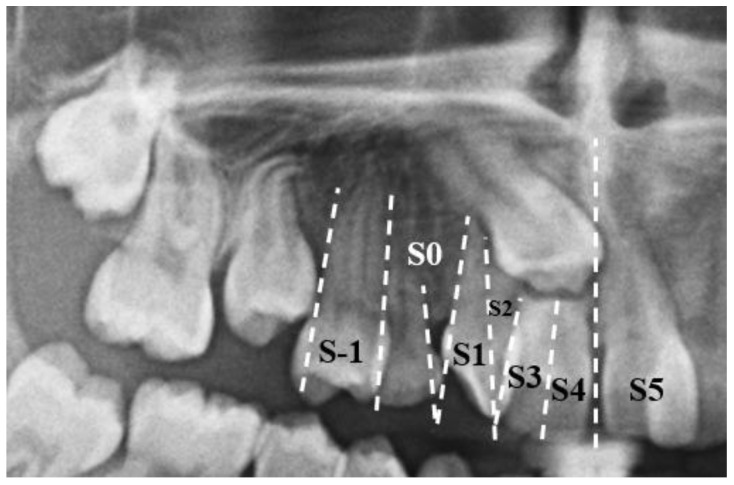
Sectors 1–5 (mesiodistal (MD) position of the maxillary canine crown in relation to adjacent teeth).

To assess impaction severity, 3D diagnostic measurements were conducted, including linear distance (d1), sector analysis (sectors 1–5), and α-angulation [27]. The impacted canine presented with a steep axial angulation of 45°, significantly greater than that of normally erupted canines (~11°), and was situated high in the alveolar process with rotation. Sector analysis confirmed a severe mesial position, aligning with the extensive resorption observed in tooth #11 (Figure 11, Figure 12 and Figure 13).

**Figure 14:**
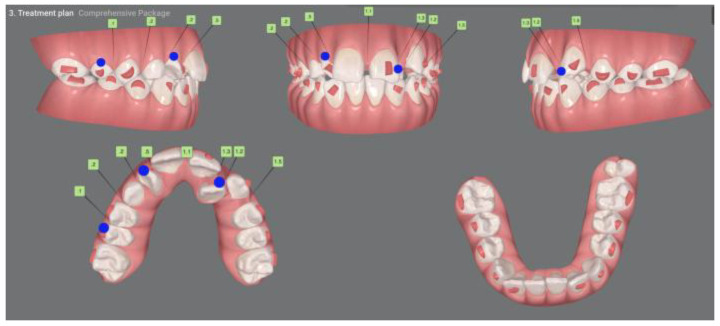
Initial ClinCheck planning.

**Figure 15:**
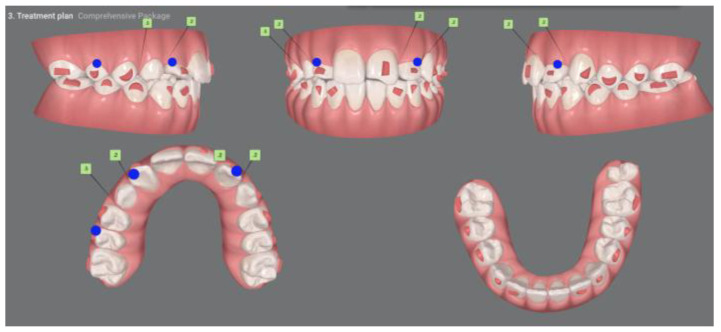
Clincheck in the final stage of the treatment plan.

**Figure 16:**
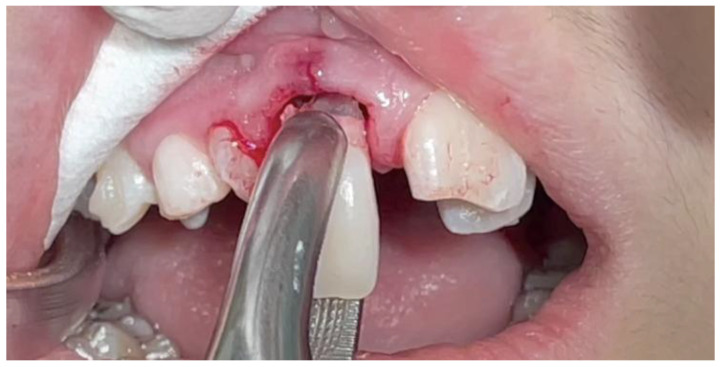
Tooth #11 extraction.

Given the severity of the findings, a multidisciplinary treatment plan was formulated: extraction of tooth #11, orthodontic traction of the impacted canine (#13), and future restorative remodeling. Digital planning was performed using the ClinCheck^®^ Pro software, version 6.0, ensuring no significant movement of the root of tooth #12, particularly during the initial stages, to avoid interference with canine traction (Figure 14 and Figure 15). Due to the advanced resorption and mobility, tooth #11 was extracted (Figure 16).

**Figure 17:**
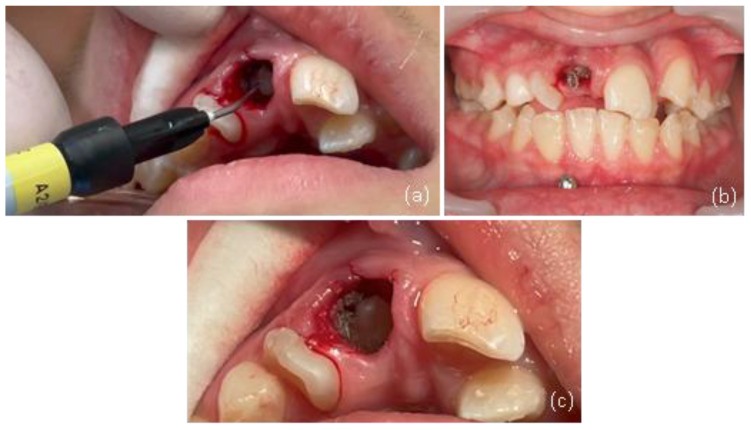
(**a**–**c**) Bonding of button with a stainless steel ligature wire using the sandwich technique and flowable resin in the extraction site of tooth #11.

**Figure 18:**
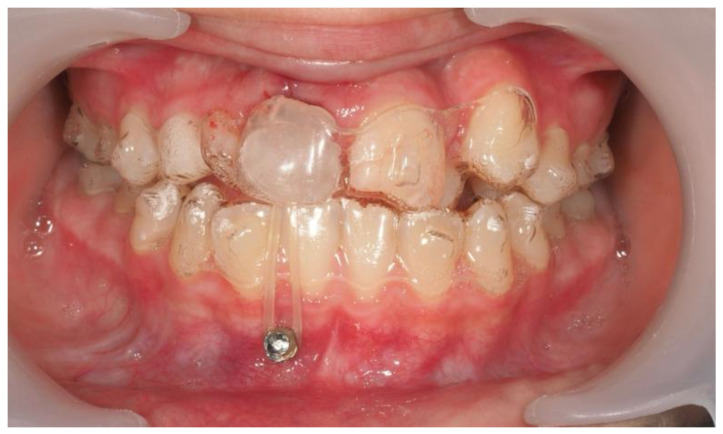
Temporary skeletal anchorage devices (TADs) placed between the lower central and lateral incisors to aid traction of tooth #13. Orthodontic wax shaped as tooth #11 inserted into the aligner for aesthetic camouflage during treatment.

A traction button was bonded within the extraction socket using the “sandwich technique,” which involves securing a stainless steel ligature wire between two layers of flowable resin (Figure 17) [28]. This provided stable anchorage in a restricted area. Temporary anchorage devices (TADs) were placed between the lower incisors to assist vertical traction. For aesthetics, a wax pontic replicating tooth #11 was inserted into the aligners (Figure 18).

**Figure 19:**
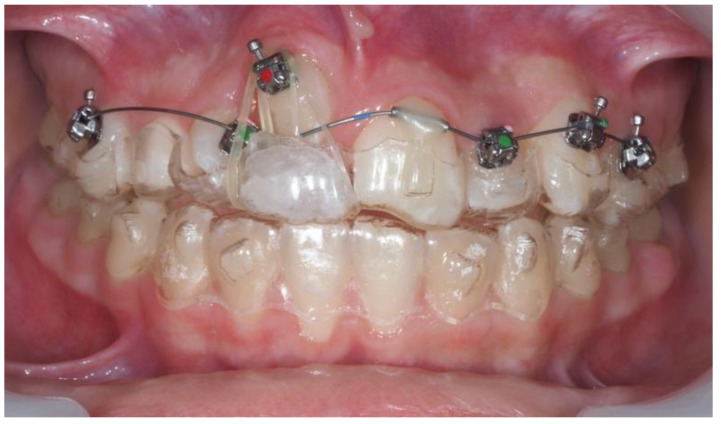
Sectional fixed appliance and elastic traction setup through the aligner.

Following surgical exposure and the eruption of tooth #13 into the extraction space, a sectional fixed appliance was placed on teeth #14, #12, #22, #23, and #24, with a microtube on #21. Elastics were used from the bracket on tooth #13 and were routed through the pontic for continued traction. Aligners were used during meals for aesthetics. Both arches were treated with aligners during this transitional stage (Figure 19).

**Figure 20:**
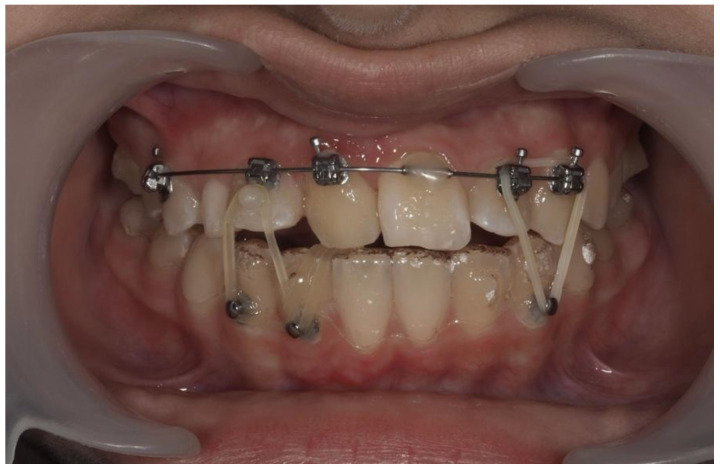
Triangular and inverted triangular elastics for midline correction.

As traction advanced, upper aligners were temporarily paused during the final stages of canine repositioning to prevent interference, while lower aligner treatment continued. Buttons were placed on teeth #42 and #43; an additional button was bonded more cervically on #12 to aid vertical control. Triangular and inverted triangular elastics were applied between teeth #22, #23, and #33 to close the bite and refine the midline (Figure 20).

**Figure 21:**
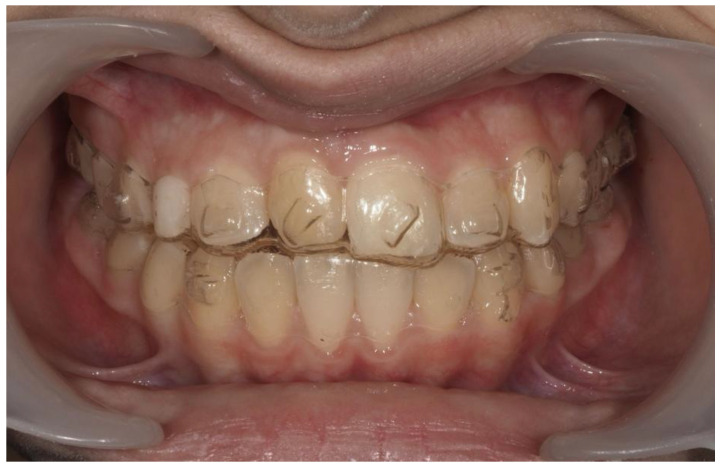
Additional aligners for occlusion improvement.

**Figure 22:**
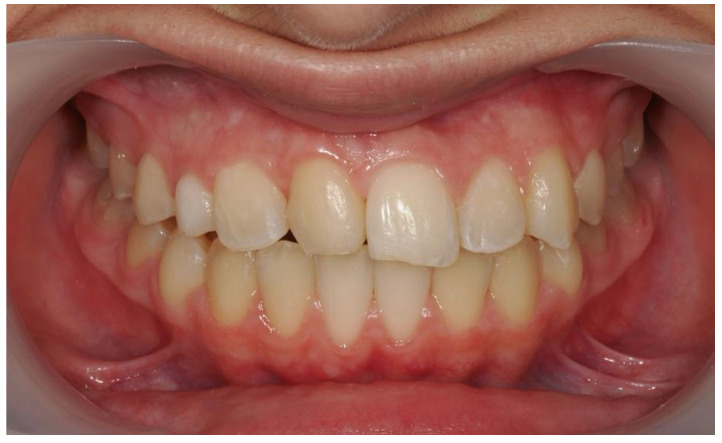
Final intraoral image showing correct overjet and overbite.

**Figure 23:**
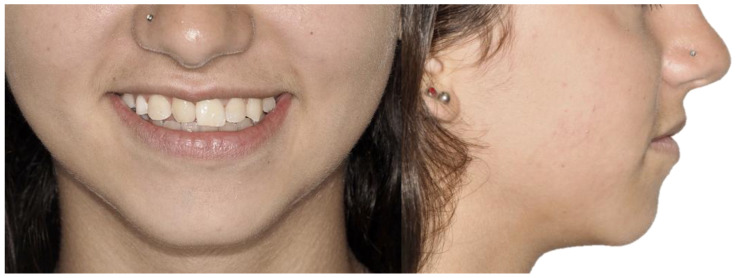
Final smile of tooth #13 in position #11 and profile view.

**Figure 24:**
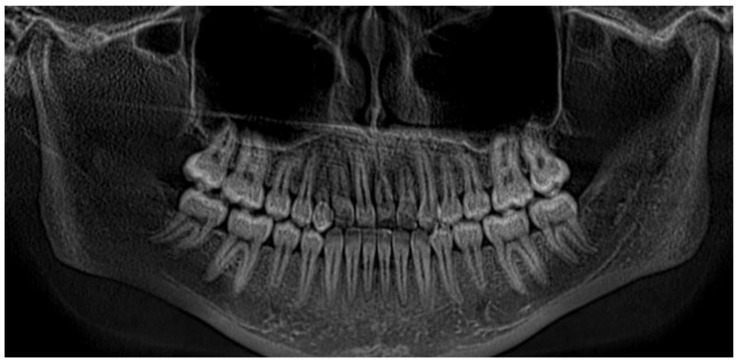
Final x-ray unveiling the position of tooth #13 in the site of tooth #11.

**Figure 25:**
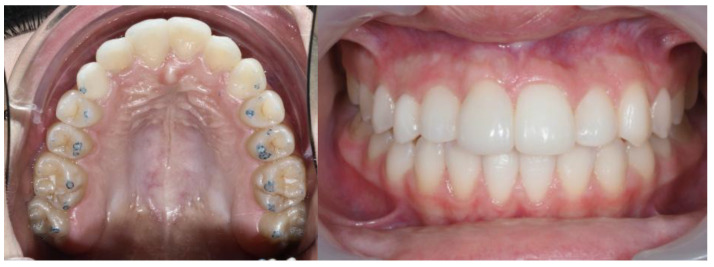
Composite build-up of tooth #13 and aesthetic reshaping of tooth #53.

Additional aligners were planned to finalize occlusion (Figure 21). Once tooth #13 reached the position of the extracted central incisor (#11), and the ideal overjet and overbite were achieved, composite resin was used to reshape #13 with central incisor morphology. Resin build-up was also applied to tooth #53 to improve aesthetics (Figure 22, Figure 23, Figure 24 and Figure 25).

**Figure 26:**
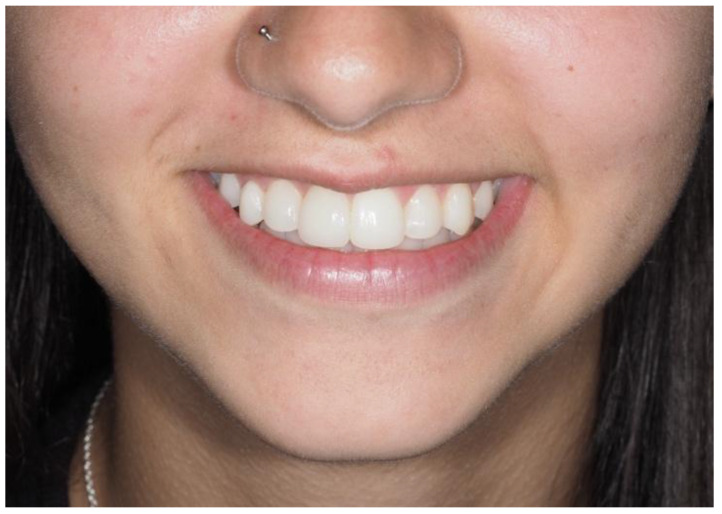
Final smile showing harmonious and aesthetic result.

After three years of treatment, the final results validated the chosen strategy. Tooth #13 was successfully relocated to the central incisor position (#11), tooth #12 was preserved and properly aligned despite its unfavorable root angulation, and tooth #53 remained functional. The case concluded with stable occlusion and an aesthetically pleasing, harmonious smile (Figure 26).

## 4. Discussion

The findings of this narrative review provide valuable insights into the clinical management of maxillary canine transposition (MCT), a rare and challenging dental anomaly. Across the 48 cases reviewed, including both retrospective studies and case reports, a majority (68.7%) underwent orthodontic correction [6,9,10,11,13,14,15,16,18,19,22,23,24], while 31.3% were maintained [7,8,9,10,12,17,20,21]. These results suggest that correction is generally favored when feasible, yet the choice depends on several factors, notably the type and complexity of the transposition.

Canine–lateral incisor transpositions (Mx.C.I2) were most frequently corrected (86.7%) [10,11,14,15,18,19,22,24], reflecting their relatively favorable anatomical configuration. Incomplete transpositions also showed a high correction rate (89%) [6,9,11,15], likely due to simpler biomechanics compared to complete transpositions. Conversely, complete transpositions had lower correction rates (61.1%) [6,13,14,15,16,18,19,22,23,24], emphasizing the influence of anatomical constraints.

The data also revealed that malocclusion type influences treatment decisions. Class II cases were corrected more often (71.4%) [6,14,16,22], while all Class III and Mx.C.I1 cases were maintained [8,17,21]. Similarly, the eruption pattern was a decisive factor; vestibularly impacted canines were corrected in 85.7% of cases [13,14,15,16,19,22,24], while all palatally impacted canines were maintained [7,8,20], indicating the surgical and mechanical complexity associated with palatal positioning.

The clinical case presented exemplifies the challenges of managing a highly complex maxillary canine–central incisor transposition (Mx.C.I1), which was diagnosed in a 13-year-old female. The imaging revealed total root resorption of tooth #11 caused by intrusion of the transposed canine (#13), along with severe horizontal displacement of the lateral incisor (#12), which was located posterior to the canine crown. These anatomical complications rendered corrective transposition unfeasible.

Given the irreversible damage and spatial limitations, a conservative approach was adopted. Tooth #11 was extracted, and controlled orthodontic traction repositioned tooth #13 into the central incisor site, followed by aesthetic reshaping with composite resin. Digital planning (ClinCheck), skeletal anchorage (TADs), and a combination of aligners and sectional fixed mechanics were employed to preserve periodontal health and ensure functional and aesthetic success.

This management strategy, emphasizing individualized planning and biological respect, aligns with findings from the narrative review, where all Mx.C.I1 cases were maintained due to their inherent complexity. The successful outcome in this case supports the rationale for maintaining transpositions in similarly challenging scenarios, reinforcing the need for early diagnosis, multidisciplinary coordination, and personalized treatment protocols.

Several limitations must be acknowledged. The small number of included studies (only two retrospective) limits the generalizability of the conclusions [9,10]. Both are retrospective and supplemented by case reports, which are prone to bias and lack standardized outcomes. The absence of prospective studies also precluded meta-analysis or quantification of effect size. The risk of bias was high due to inconsistent reporting, selection bias, and methodological heterogeneity. These limitations highlight the need for well-designed prospective studies to better inform clinical decision-making.

This narrative review and case report emphasize the multifactorial nature of MCT and the importance of individualized planning. By documenting a rare and anatomically severe case of Mx.C.I1 where the transposition was maintained rather than corrected, we reinforce the value of conservative strategies in complex clinical scenarios. Despite the rarity of this condition, our findings demonstrate that, with careful planning and interdisciplinary collaboration, even highly atypical cases can achieve functional and aesthetic success.

## 5. Conclusions

Maxillary canine transposition is a rare anomaly with no standardized treatment protocol. Clinical decision-making should be guided by anatomical conditions, eruption pattern, malocclusion classification, and the feasibility of achieving safe biomechanical movement.

The narrative review indicates that correction is preferred in most cases—especially in canine–lateral incisor and incomplete transpositions—but maintenance may be more appropriate in anatomically complex scenarios, such as Mx.C.I1 or palatal impactions.

The presented case confirmed this trend, showing that maintenance of the transposed canine, combined with interdisciplinary planning and conservative strategies, can lead to satisfactory outcomes even in severe cases.

Until stronger evidence is available, the treatment of MCT should remain case-specific, using digital planning and early diagnosis to guide biologically sound decisions.

## Figures and Tables

**Table 1:** PICOS Strategy applied to the current review.

P	Patients with at least one transposed maxillary canine
I	Analyze multiple treatments of maxillary canine transposition
C	Compare maxillary canine transposition cases
O	Conclude when the maxillary canine transposition should be corrected or maintained
S	Retrospective studies and case reports

**Table 2:** Databases and research strategy.

Data Bases	Advanced Research	Articles
PubMed	(cuspid [MeSH Terms] OR (cuspid) OR (cuspids) AND (maxilla[MeSH Terms]) AND (tooth eruption, ectopic [MeSH Terms]) OR (ectopic tooth eruption) OR (tooth eruptions, ectopic) OR (ectopic tooth eruptions)	134
ScienceDirect	(ectopic tooth eruption AND maxilla AND tooth eruption, ectopic AND orthodontics)	112
Cochrane Library	(ectopic tooth eruption AND maxilla AND tooth eruption, ectopic AND orthodontics)	15
EBSCO	(cuspid) AND (maxilla) AND (tooth eruption, ectopic) OR (ectopic tooth eruption) OR (tooth eruptions, ectopic) OR (ectopic tooth eruptions) AND (orthodontics)	170
Scopus	(ectopic tooth eruption AND maxilla AND tooth eruption, ectopic AND orthodontics)	22

## Data Availability

Data that support this study’s findings are available from the corresponding author upon request.

## Abbreviations

The following abbreviations are used in this manuscript:

Mx.C.P1	Maxillary canine–first premolar transposition
Mx.C.I2	Maxillary canine–lateral incisor transposition
Mx.C.M1	Maxillary canine–first molar transposition
Mx.I2.I1	Maxillary central incisor–lateral incisor transposition
Mx.C.I1	Maxillary canine–central incisor transposition
CBCT	Cone beam computed tomography
IMC	Impacted maxillary canines
MCT	Maxillary canine transposition
PICOS	Population/Patient/Problem; Intervention; Comparation; Outcomes; Study design
